# Unhealthy Houses

**Published:** 1862-06

**Authors:** 


### UNHEALTHY HOUSES.

A man requires ten cubic feet of air every minute, in order to supply an amount of oxygen sufficient for the wants of the system. The air enters the lungs full of oxygen, it leaves them without an atom, hence that which leaves the lungs is so wholly unfit for breathing purposes, that if re-breathed, unmixed with any other air whatever, it would cause instant suffocation. This unnutritious air is so very light that when out of doors it rises instantly toward the clouds, as may be seen any frosty morning; but when a person is in a close room, this unwholesome air mixes with that which surrounds it, and in a few moments the whole air of any room becomes contaminated, and this contamination becomes more aggravated, more virulent at every breath, and this is the reason of the greater rapidity with which persons and animals recover from terrible wounds when they have to lie out in the open air, with nothing but water to drink and roots or berries to eat for days together, when in fact they would certainly have died if they had had all the comforts of home, fireside, and friends. It is estimated that in the course of fifty years, a man, in round numbers, breathes five hundred millions of times, taking into his lungs one hundred and seventy tons weight of air, and discharging therefrom twenty tons of deadly carbonic acid gas. The great aim of those who have an ambition to live long and healthfully should be to rebreathe as little of this pernicious gas as possible, and to have as many as practicable, of the five hundred millions of breaths to be drawn, to be taken from the exhaustless store-house of "all out-doors." If the circumstances of one's life should make it necessary to spend a large portion of existence in-doors, then it should be a constant aim and study to have a good ventilation, to facilitate the egress of the bad air, and that its place should be supplied by that which is pure, invigorating, and life-giving.

Cases are given to show that a malign influence has been given to rooms, houses, and circumscribed out-door localities; influences so potent, so invisible, so persistent, and so mysterious, as to inspire an indefinable dread and awe of the place. Napoleon the First once ordered the destruction of a sentry-box in which several soldiers had successively committed suicide. A case was reported officially lately, in Paris, to the effect that a gentleman, without any known reason, destroyed himself; that the person who occupied the apartment before him, and also the latter's predecessor, had committed suicide.

A correspondent writes, April 11th, 1862:

"Our house is situated on the bank of a large river, on rather low ground, with salt-marshes on one side. So much for the outside. The inside is the great bugbear. A large drain runs through the middle of the house, from front to rear, and through the lawn to the river.

"On either side of the front of the house is a cistern and pump of spring-water, waste-water from both running through this drain, which is directly under the flooring of the kitchen-entry, there being no sub-cellars.

"At the river-end of this lower entry are the two water-closets, contents passing to the river through said drain.

"Now the stench (for it is nothing else) through the house from this sewer is overpowering at times, and always disagreeable. When the wind blows from the river, words can give no idea of the effluvia.

"Before I investigated the subject I was afflicted for years with a constant diarrhea, for which I consulted several physicians, and adopted several kinds of treatment.

"My sister, who slept with me, had three long illnesses of bilious fever. She slept in the corner of our room, just over the drain. The smell in that spot is intense. The bedstead was then removed as far as possible from the noxious corner, plenty of sunshine let in the room, and we have found much benefit from so doing.

"A relative, who was never sick in his life before he moved into this house, has had fever and ague several times; and I am convinced that if it were not that my mother and her four daughters almost live in the open air—riding, driving, walking, and gardening—they would be confirmed invalids from the air generated through this horrid drain."

The gas which lights our dwellings, even when pure, causes great contamination, unless the products of combustion are speedily conveyed away. But illuminating gas is never pure, it always contains bisulphide of carbon, its burning yields sulphurous acid, which speedily becomes oil of vitriol; another product is sulphurated hydrogen.

If a man weighs himself at bedtime, and again on first rising, he will find an actual loss in weight of half a pound, which amount has gone off from his body and has been distributed through the bed-clothing and the air of the room. If a single ounce of old woolen rags is burned in a chamber, the atmosphere becomes impregnated with the smoke and is scarcely endurable, yet sixteen times that much of foreign material, of dead and refuse parts of the body, are mixed with the air of a chamber, and although not producing so ill an odor, make it sixteen times more injurious, because the air is just sixteen times more impure, has sixteen times less of the appropriate nourishment of the system, showing again the great importance of sleeping in well-ventilated chambers. If two persons sleep in the same room, these pernicious deteriorations of the atmosphere are doubled.

